# OPV Vaccination and Shedding Patterns in Mexican and US Children

**DOI:** 10.1093/cid/ciy636

**Published:** 2018-10-30

**Authors:** Jonathan Altamirano, Clea Sarnquist, Rasika Behl, Lourdes García-García, Leticia Ferreyra-Reyes, Sean Leary, Yvonne Maldonado

**Affiliations:** 1 Stanford University School of Medicine, California; 2 Instituto Nacional de Salud Pública, Cuernavaca, Morelos, Mexico

**Keywords:** oral poliovirus vaccine, poliovirus, shedding patterns, Mexico, United States

## Abstract

**Background:**

As wild poliovirus is eradicated and countries switch from oral poliovirus vaccine (OPV) to inactivated poliovirus vaccine (IPV) per World Health Organization recommendations, preventing circulation of vaccine-derived poliovirus (cVDPV) is a top priority. Currently, the impact of prior poliovirus vaccination on OPV shedding is not fully understood.

**Methods:**

Stool samples from 2 populations were tested for OPV to assess shedding patterns. 505 samples from 43 US children vaccinated with OPV were collected over 42 days post-vaccination. 1,379 samples from 148 Mexican children vaccinated with OPV were collected over 71 days post-vaccination. Prior vaccination history was recorded for both groups.

**Results:**

Seventeen (40%) of the US children had never received poliovirus vaccination while the Mexican children had received at least 2 doses of IPV and 116 (78%) had OPV exposure. In total, 84% of US children and 78% of Mexican children shed OPV (P = .44, Fisher exact test), with a mean shedding duration of 17.4 days for US children and 9.3 days for Mexican children (P < .0001, Wilcoxon-Mann Whitney test).

**Conclusions:**

Prior vaccination did not affect the likelihood of shedding, as the US and Mexico cohorts had similar shedding proportions. However, prior vaccination affected shedding duration as the Mexican children, who were largely OPV exposed and all of whom had at least 2 IPV vaccinations, shed OPV for half as long as the US cohort. Since different countries maintain different poliovirus vaccination schedules, it is likely that duration of shedding of OPV varies in populations around the world.

In 1988, the World Health Organization announced the inception of the Global Polio Eradication Initiative, dedicated to the eradication of wild poliovirus (WPV). Since then, polio eradication efforts have successfully decreased the number of WPV cases by more than 99%, from 350000 worldwide in 1988 to only 22 reported cases in 2017 [1, 2]. WPV serotype 1 is currently believed to be the only serotype still endemic in Pakistan and Afghanistan, while WPV serotype 2 was declared eradicated in September 2015, and WPV serotype 3 has not been detected since November 2012 [1, 3].

Widespread vaccination with Sabin oral poliovirus vaccine (OPV) is largely responsible for the success of polio eradication efforts. OPV is particularly effective in low- and middle-income settings due to its low cost, easy administration, and ability to provide community-level immunization through fecal–oral transmission from vaccinees to household and community contacts [4]. However, continued use of OPV will continue to cause cases of vaccine-associated paralytic polio (VAPP), and prolonged circulation of OPV can result in the development of neurovirulent vaccine-derived polioviruses (VDPV). VAPP is estimated to cause 2–4 cases of paralysis per 1000000 live births per year in countries using OPV [5]. Circulating VDPVs (cVDPVs), genetically divergent vaccine viruses with evidence of community transmission, have been shown to cause paralysis that is indistinguishable from paralysis caused by WPV [6]. In 2017, 91 cases of cVDPVs were identified, particularly after outbreaks of cVDPV serotype 2 in Syria and the Democratic Republic of Congo, which were >4 times the number of WPV cases identified in 2017 [2].

The Polio Eradication and Endgame Strategic Plan 2013–2018 established by the Global Polio Eradication Initiative proposes the introduction of IPV into routine childhood vaccination in countries using only OPV and the eventual removal of OPV from global use [7]. This transition began in April 2016 and May 2016 with the replacement of trivalent OPV (tOPV), which contains OPV serotypes 1, 2, and 3, with bivalent OPV (bOPV), which does not contain OPV serotype 2 [3]. Stockpiles of monovalent OPV-2 have been prepared in the event of future outbreaks of cVDPV serotype 2 [7, 8].

With this in mind, understanding the impact of prior polio vaccination and immunity on OPV shedding in OPV-vaccinated children is of critical importance. Immune responses to OPV and IPV have been extensively studied in a variety of settings. OPV has been shown to produce both humoral and mucosal immunity [9–12]. By contrast, while IPV has been shown to provide humoral immunity, there is limited evidence that it provides mucosal immunity to poliovirus [12–14]. The exception to this appears to be IPV vaccination after prior exposure to live poliovirus, which seems to boost mucosal immunity [14].

In order to better understand the impact of different polio vaccination schedules on OPV shedding patterns in vaccinees, we compared stool samples obtained after OPV vaccination in 2 distinct populations: children living in Palo Alto, California, in 1998 before OPV cessation in the United States, and Mexican children from 3 rural communities in Orizaba, Veracruz, during a National Health Week (NHW). We chose to compare OPV shedding patterns in these 2 populations as we have a unique dataset from these populations with distinct poliovirus vaccination schedules.

## METHODS

The data used in this analysis were collected for 2 distinct OPV studies [15–17]. The first was an analysis of samples after routine OPV administration looking at the acquisition of a canonical point mutation in OPV-3 known to be associated with OPV-3 VAPP, conducted from February 1998 to December 1998 in Palo Alto, California [17]. Parents of 1-year-old children were asked to collect 11 stool samples: 1 baseline sample, 1 sample/day for 1 week after OPV vaccination, and 1 sample at the end of the second, fourth, and sixth week (days 14, 28, and 42) after OPV vaccination. The parents of most children provided additional samples during the second week after vaccination (days 8–13). Vaccination records and primary household languages were collected for 51 children, and 43 children provided a total of 505 samples, which were included in this analysis. In the original analysis, only those 28 children with prior IPV vaccination were included.

The second study was a prospective cluster-randomized trial in 3 rural indigenous Mexican communities in Orizaba, Veracruz, Mexico (Capoluca, Campo Grande, and Tuxpanguillo) [15–16]. Approximately 150 households were enrolled in each community, with each household having at least 1 child aged <5 years with an up-to-date IPV vaccination record who was eligible to receive OPV. Each community then received a different level of OPV coverage during the Mexican NHW in February 2015: 70% of enrolled households in Capoluca, 30% in Campo Grande, and 10% in Tuxpanguillo. A total of 155 children were randomly selected to receive OPV, with no other children in these communities receiving OPV until the May 2015 NHW. After enrollment, 10 stool samples were scheduled for collection from each member of all enrolled households: 1 baseline sample collected 2–3 weeks before vaccination and then samples collected 1, 4, 7, 10, 14, 21, 28, 51, and 71 days after vaccination. In total, 148 vaccinees provided 1379 samples, which were included in this analysis. We also collected demographic and health information from all participants via household surveys.

Stool samples from both studies were analyzed for OPV isolates using quantitative reverse transcription polymerase chain reaction (RT-qPCR). Samples were run in triplicate and considered positive for OPV if two-thirds of the reactions had a threshold cycle <37. Positive samples were Sanger sequenced, when possible, to confirm results. More details about the protocol for this analysis are provided by Van Hoorebeke et al [18].

Shedding duration was calculated with the assumption that a vaccinee’s first positive sample was the first day that vaccinees shed OPV, and that their last positive sample was the last day that vaccinees shed OPV. Additionally, shedding durations were only calculated if participants had multiple positive samples with less than 1 week between positive samples. Comparisons of shedding duration, both overall and by OPV serotype, between US and Mexican vaccinees were done for the overall groups, including all vaccinees, and for the IPV-vaccinated, OPV-naive vaccinees. For Mexican vaccinees, the shedding duration of OPV-exposed vaccinees was compared to OPV-naive vaccinees. For US vaccinees, the shedding duration of poliovirus vaccine-naive children was compared to that of IPV-vaccinated children. These comparisons were made using Wilcoxon-Mann-Whitney test. Comparisons of the number of vaccinees who shed OPV, both overall and by each OPV serotype, as well as for the number of vaccinees with multiple positive samples were made using Fisher exact test. A *P* value of <.05 was considered statistically significant.

## RESULTS

The vaccination history of the vaccinees is described in Table 1. All Mexican vaccinees had at least 2 doses of IPV, and most vaccinees had 4 doses of IPV (71%) prior to the study period. The majority of Mexican vaccinees had also been previously exposed to OPV (78%). By contrast, most of the US vaccinees were OPV naive (91%) and were largely divided into 2 groups: participants with no prior polio vaccination (40%) and participants with 2 IPV doses (47%).

**Table 1.** Vaccinee Demographics

Demographics	Mexico	United States
Number of vaccinees	148	43
Number of shedding vaccinees	116 (78%)	36 (84%)
OPV-1 positive vaccinees	87 (59%)	32 (74%)
OPV-2 positive vaccinees	97 (66%)	32 (74%)
OPV-3 positive vaccinees	73 (49%)^a^	34 (79%)^a^
Vaccinees with multiple positive samples	93 (80%)	34 (94%)
Vaccination history		
No prior vaccination	…	17 (40%)
IPV only	32 (22%)	22 (51%)
1 IPV	…	2 (5%)
2 IPV	1 (1%)	20 (47%)
3 IPV	19 (13%)	…
4 IPV	12 (8%)	…
OPV exposed	116 (78%)	4 (9%)
OPV only	...	3 (7%)
IPV/OPV	116 (78%)	1 (2%)
1 IPV	...	...
2 IPV	...	1 (2%)
3 IPV	23 (16%)	...
4 IPV	93 (63%)	...
Number of samples tested	1379	505
Number of positive samples	373 (27%)	316 (63%)
OPV-1 positives	205 (55%)	195 (62%)
OPV-2 positives	263 (71%)	237 (75%)
OPV-3 positives	211 (57%)	211 (67%)

Abbreviations: IPV, inactivated poliovirus vaccine; OPV, oral poliovirus vaccine.

^a^ *P* < .01 using Fisher exact test.

A total of 116 (78%) Mexican vaccinees and 36 (84%) US vaccinees shed OPV after vaccination (Table 1). While a higher proportion of the US vaccinees shed OPV, this difference was not statistically significant (*P* = .44). For each serotype, a higher proportion of the US vaccinees were found to shed each serotype: Mexico vs US OPV-1, 59% vs 74%; OPV-2, 66% vs 74%; and OPV-3, 49% vs 79%. Of these differences, only OPV-3 was statistically significant (*P* = .0005). Most vaccinees in both groups also shed at multiple time points throughout the studies, with 80% of Mexican vaccinees and 94% of US vaccinees shedding at least twice during the study periods. This difference was not statistically significant (*P* = .07).

Shedding durations were calculated for each group overall, as well as for each distinct polio vaccine combination (Table 2). Generally, in both populations, OPV-1 had the shortest shedding duration, with OPV-2 either being very similar or slightly longer, and OPV-3 having the longest shedding duration. Looking at the overall data, the US vaccinees shed significantly longer than the Mexican vaccinees, 17 vs 9.3 days (*P* < .0001). By serotype, the US vaccinees also shed longer than the Mexican vaccinees for all serotypes: 7.7 vs 7.1 days for OPV-1, 10.1 vs 7.3 days for OPV-2, and 17.4 vs 11 days for OPV-3. The differences for OPV-2 and OPV-3 were both statistically significant (*P* = .01 and *P* = .02, respectively), while the difference in duration for OPV-1 was not significant (*P* = .62). In the US vaccinees, both overall and across serotypes, the IPV-vaccinated children shed slightly longer than the children with no prior polio vaccination: 19.4 vs 17 days overall, 8.2 vs 5.7 days for OPV-1, 11.7 vs 7.9 days for OPV-2, and 20.4 vs 18.1 days for OPV-3 (Table 2). These differences were not statistically significant. In the Mexican vaccinees, OPV-exposed children shed significantly less than OPV-naive children, both overall and across all 3 serotypes: 7.6 vs 14.3 days (*P* = .0004) overall, 5.7 vs 10.3 days (*P* = .03) for OPV-1, 5.7 vs 10.3 days (*P* = .0002) for OPV-2, and 7.8 vs 15.3 days (*P* = .0015) for OPV-3. Additional subgroups are listed in Supplementary Table S1.

**Table 2.** Shedding Durations by Location and Previous Vaccination

	Mexico Vaccinees								US Vaccinees						
	Overall								Overall						
Variable	N	Mean	Median	SD	SE	Min.	Max.	Variable	N	Mean	Median	SD	SE	Min.	Max.
Overall duration^a^	94	9.3	6.0	7.1	0.7	2	29	Overall duration^a^	34	17.4	17.0	10.8	1.8	2	42
OPV-1 duration	54	7.1	6.0	5.3	0.7	2	29	OPV-1 duration	29	7.7	7.0	5.3	1.0	1	21
OPV-2 duration^b^	72	7.3	6.0	5.4	0.6	2	27	OPV-2 duration^b^	31	10.1	9.0	6.2	1.1	1	25
OPV-3 duration^b^	49	11.0	8.0	7.4	1.1	2	29	OPV-3 duration^b^	29	17.4	17.0	11.8	2.2	1	42
	No Prior Polio Vaccination								No Prior Polio Vaccination						
Overall duration	…	…	…	…	…	…	…	Overall duration	12	17	17.0	10.8	3.1	2	42
OPV-1 duration	…	…	…	…	…	…	…	OPV-1 duration	9	5.7	3.0	6.1	2.0	2	21
OPV-2 duration	…	…	…	…	…	…	…	OPV-2 duration	11	7.9	8.0	6.3	1.9	1	19
OPV-3 duration	…	…	…	…	…	…	…	OPV-3 duration	11	18.1	17.0	10.5	3.2	2	42
	IPV-Only Vaccination								IPV-Only Vaccination						
Overall duration^c^	24	14.3	12.0	8.4	1.7	3	29	Overall duration	18	19.4	18.5	10.9	2.6	5	40
OPV-1 duration^d^	17	10.3	9.0	7.5	1.8	3	29	OPV-1 duration	17	8.2	10.0	4.0	1.0	1	15
OPV-2 duration^c^	24	10.3	9.0	6.0	1.2	3	24	OPV-2 duration	17	11.7	11.0	6.1	1.5	1	25
OPV-3 duration^c^	21	15.3	17.0	7.7	1.7	3	29	OPV-3 duration	14	20.4	19.5	12.3	3.3	2	40
	OPV Exposed								OPV Exposed						
Overall duration^c^	70	7.6	6.0	5.7	0.7	2	27	Overall duration	4	9.8	8.0	7.9	4.0	3	20
OPV-1 duration^d^	37	5.7	6.0	3.1	0.5	2	14	OPV-1 duration	3	11.0	11.0	9.0	5.2	2	20
OPV-2 duration^c^	48	5.7	4.5	4.3	0.6	2	27	OPV-2 duration	3	8.7	10.0	5.1	3.0	3	13
OPV-3 duration^c^	28	7.8	6.5	5.3	1.0	2	27	OPV-3 duration	4	4.8	3.5	4.3	2.2	1	11

Abbreviations: IPV, inactivated poliovirus vaccine; OPV, oral poliovirus vaccine; SD, standard deviation; SE, standard error.

^a^ *P* < .0001 using Wilcoxon-Mann-Whitney test across populations. Comparisons made overall and for IPV-only vaccination.

^b^ *P* < .05 using Wilcoxon-Mann-Whitney test across populations. Comparisons made overall and for IPV-only vaccination.

^c^ *P* < .01 using Wilcoxon-Mann-Whitney test between vaccine schedules within populations. Comparisons made for: Mexican vaccinees—IPV-only vs OPV-exposed; USA—no prior vaccination vs IPV-only vaccination.

^d^ *P* < .05 using Wilcoxon-Mann-Whitney test between vaccine schedules within populations. Comparisons made for: Mexican vaccines—IPV-only vs OPV-exposed; USA—no prior vaccination vs IPV-only vaccination.

Most vaccinees who shed had OPV isolates in their samples by day 4 in both populations. For Mexican vaccinees 41% began to shed 1 day after OPV vaccination, and an additional 48% were shedding OPV by day 4. Similarly, 69% of the US vaccinees were shedding within 3 days of OPV vaccination, with an additional 22% beginning to shed before the end of the first week after vaccination. Additionally, for both populations, most vaccinees who began to shed early in the study period also had multiple positive samples throughout the study. While few vaccinees began shedding after the first week, the Mexican vaccinee data suggest that vaccinees who began to shed later were less likely to shed OPV for extended periods of time, as shown by a decrease in the percent of vaccinees who shed OPV in multiple samples.

The serotype shedding patterns appeared to be similar for both populations. OPV-1 peaked the earliest, by days 4 and 3 for the Mexican and US vaccinees, respectively. OPV-1 was the second most isolated serotype until day 7 for the Mexican vaccinees and until day 10 for US vaccinees, at which point it was the least detected OPV serotype. OPV-2 became the dominant isolate found in samples by day 4 and peaked at day 7 and day 9 for the Mexican and US vaccines, respectively. Additionally, OPV-2 was the most commonly found isolate in both groups and was largely responsible for the peak of overall shedding, also at days 7 and 9. After peak shedding, OPV-2 showed a sharp decline, becoming the second most detected serotype. The 2 groups showed the largest difference in OPV-3 shedding. In the Mexican vaccinees, peak OPV-3 shedding occurred at day 7, after which OPV-3 declined slowly for the rest of the study period. In contrast, the US vaccinees demonstrated sustained shedding for OPV-3, peaking at day 4 and declining after day 14. However, in both groups, OPV-3 was found in the lowest quantities for the first 7–10 days of the study and became the dominant serotype isolated from samples by day 14.

## DISCUSSION

Here, we present the results of RT-qPCR analysis of 1884 stool samples collected from 2 distinct populations: 1379 samples collected in 2015 from 148 Mexican vaccinees who were primarily IPV vaccinated and had prior OPV exposure and 505 samples collected in 1998 from US vaccinees who were either completely unvaccinated or primarily IPV vaccinated. Prior poliovirus vaccination history did not significantly impact the likelihood of OPV shedding in these populations, as a similar proportion of vaccinees in both countries had OPV isolates in their stool. Prior literature has found OPV vaccination, as well as OPV-IPV combined vaccination, to be protective against shedding after a dose of OPV [10–12, 14]. However, it is possible that this protective effect wanes over time. OPV is only provided twice annually in Mexico, in February and May. As the study period in Mexico began in February 2015, the last time these children were exposed to OPV before the study began was May 2014. This suggests that the protective effect of OPV exposure against the likelihood to shed OPV wanes within 9 months.

Although prior vaccination did not appear to significantly impact the likelihood to shed, certain vaccination combinations did appear to impact duration of OPV shedding. Overall, the Mexican children had significantly lower overall shedding durations than the US vaccinees and also had significantly lower shedding durations for OPV-2 and OPV-3. This decreased shedding duration indicates that there may be increased mucosal immunity against OPV [12, 14] and is likely the result of OPV exposure in the Mexican vaccinees that is absent among most of the US vaccinees. This conclusion is supported by our data in several ways. First, our data shows no significant difference between the IPV-only vaccinees in both populations, indicating that the differences overall were not due to this subgroup. Second, there were no significant differences in the US vaccinees when comparing the unvaccinated OPV recipients and the IPV-only vaccinees. These results are also supported by prior literature, which has shown that IPV does not provide mucosal immunity and that OPV–IPV combinations provide higher levels of mucosal immunity [14].

Shedding patterns in both populations were similar for OPV-1 and OPV-2, indicating that the differences in prior polio vaccination may not impact serotype-specific shedding patterns. However, differences in OPV-3 shedding were detected between the 2 populations, as the US vaccinees demonstrated longer sustained shedding of OPV-3 up to 14 days after vaccination, which was not found in the Mexican vaccinees. This difference could reflect the variability in sample collection, which occurred every 3 days for the first 2 weeks of the study among the Mexican vaccinees, as opposed to the daily samples that were collected for the US vaccinees. It is interesting to note that in the original publication of the US samples, bimodal shedding of OPV-3 was identified and significantly associated with an early single-point mutation related to OPV-3 VAPP [17]. An understanding of how genotypic factors and related acquired mutations in OPV serotypes may impact shedding dynamics is needed.

OPV-2 was the most commonly found OPV serotype, identified in 71% and 75% of all positive samples from the Mexican and US vaccinees, respectively. Prior OPV studies have also found OPV-2 to be the most commonly found isolate in stool and sewage samples, which likely contributes to the increased transmission of OPV-2 and the high prevalence of cVDPV serotype 2 [3]. With the transition to bOPV, research will need to focus on changes to shedding patterns that may occur in OPV-1 and OPV-3 in the absence of OPV-2.

Our analysis has some key limitations. First, some vaccination groups were too small for us to draw any meaningful conclusions, such as the OPV-only children, which made up 7% of the US vaccinees. Second, we cannot account for covariates that may impact shedding duration or likelihood to shed, such as sociodemographic status or anthropometric data, as they were not collected for the US vaccinees. Third, samples were not collected daily over the entire study period for all vaccinees. As a result, some shedding may have been missed between sample collections.

Our approach also has a number of strengths. First, this dataset is novel, incorporating data from 2 OPV studies in distinct populations, allowing for comparisons between unique groups. Second, in the Mexican study, researchers were able to leverage Mexico’s unique vaccination scheme to ensure that all vaccinee shedding was the result of OPV vaccination as part of our study, as no other children were vaccinated with OPV in these Mexican communities until after our study period was complete, and OPV is not used during routine childhood immunization.

In summary, we found no significant difference in the proportion of OPV shedding when comparing US and Mexican vaccinees, indicating that the differences in their prior poliovirus vaccinations did not impact their likelihood to shed OPV. However, prior poliovirus vaccination did appear to impact shedding duration, as the Mexican vaccinees, who were largely vaccinated with both IPV and OPV, shed for significantly fewer days than the US vaccinees, who either had no prior polio vaccination or who were IPV vaccinated. This difference appears to be driven by OPV exposure, as no significant differences were found in shedding duration when comparing the IPV-only vaccinees in both populations. The shedding curves for both populations were quite similar, particularly for OPV-1 and OPV-2, indicating that prior vaccination did not strongly alter the way vaccinees shed these viruses. The US children additionally showed a more sustained shedding of OPV-3, which in a previous study was associated with the acquisition of a mutation related to OPV-3 VAPP. Finally, most positive samples contained OPV-2, which may explain why most cases of cVDPVs have been serotype 2 and which supports the transition to bOPV.

With the intent to transition global childhood immunization to IPV-only vaccination schedules, more research is needed to understand what shedding patterns to expect in populations with varying plans of polio vaccination, in the event of future cVDPV outbreaks and possible reintroduction of OPV.

## Supplementary Data

Supplementary materials are available at *Clinical Infectious Diseases* online. Consisting of data provided by the authors to benefit the reader, the posted materials are not copyedited and are the sole responsibility of the authors, so questions or comments should be addressed to the corresponding author.

## Supplementary Material

Supplementary_Table_S1

## Notes

***Acknowledgments*.** The authors thank the families who participated in this study, the large field teams in Mexico that performed data and sample collection, as well as the broader team at Stanford University, including Christopher Anthony van Hoorebeke, Aisha Talib, Jennifer Lee Kang, Meira Falkovitz-Halpern, ChunHong (Stacy) Huang, and Marvin Sommer.

***Financial support*.** The Mexican study was funded by the & and Melinda Gates Foundation (grant OPP1116910).

***Supplement sponsorship.*** **This article appears as part of the supplement “Polio endgame and beyond: Vaccine choices, transmission dynamics, and surveillance implications,” sponsored by The Bill & Melinda Gates Foundation.

***Potential conflicts of interest.*** All authors: No reported conflicts of interest. All authors have submitted the ICMJE Form for Disclosure of Potential Conflicts of Interest. Conflicts that the editors consider relevant to the content of the manuscript have been disclosed.
