# Supplementary material for: OPV Vaccination and Shedding Patterns in Mexican and US Children
Source: Clin Infect Dis. 2018 Oct 30;67(Suppl 1):S85–9. doi: 10.1093/cid/ciy636 (PMC6206113; doi:10.1093/cid/ciy636)
Supplement: Supplementary_Table_S1 [file ciy636_suppl_supplementary_table_s1.docx]

| **Table S1. Shedding Durations by Location and Previous Vaccination – Additional Subsets** | | | | | | | | | | | | | | | |
| --- | --- | --- | --- | --- | --- | --- | --- | --- | --- | --- | --- | --- | --- | --- | --- |
|  | **Mexico Vaccinees** | | | | | | |  | **USA Vaccinees** | | | | | | |
|  | **OPV-only Vaccination** | | | | | | |  | **OPV-only Vaccination** | | | | | | |
| **Variable** |  |  |  |  |  |  |  | **Variable** | **N** | **Mean** | **Median** | **SD** | **SE** | **Min** | **Max** |
| **Overall Duration** |  |  |  |  |  |  |  | **Overall Duration** | 3 | 11.7 | 12.0 | 8.5 | 4.9 | 3 | 20 |
| **OPV-1 Duration** |  |  |  |  |  |  |  | **OPV-1 Duration** | 3 | 11.0 | 11.0 | 9.0 | 5.2 | 2 | 20 |
| **OPV-2 Duration** |  |  |  |  |  |  |  | **OPV-2 Duration** | 3 | 8.7 | 10.0 | 5.1 | 3.0 | 3 | 13 |
| **OPV-3 Duration** |  |  |  |  |  |  |  | **OPV-3 Duration** | 3 | 5.0 | 3.0 | 5.3 | 3.1 | 1 | 11 |
|  | **OPV-naïve; 3 IPV Doses** | | | | | | |  | **OPV-naïve; 3 IPV Doses** | | | | | | |
| **Variable** | **N** | **Mean** | **Median** | **SD** | **SE** | **Min** | **Max** | **Variable** |  |  |  |  |  |  |  |
| **Overall Duration** | 16 | 16 | 20 | 7 | 2 | 6 | 29 | **Overall Duration** |  |  |  |  |  |  |  |
| **OPV-1 Duration** | 13 | 11.8 | 9 | 8 | 2 | 3 | 29 | **OPV-1 Duration** |  |  |  |  |  |  |  |
| **OPV-2 Duration** | 16 | 11.3 | 9.5 | 6 | 1 | 3 | 24 | **OPV-2 Duration** |  |  |  |  |  |  |  |
| **OPV-3 Duration** | 15 | 15.3 | 17 | 7 | 2 | 3 | 29 | **OPV-3 Duration** |  |  |  |  |  |  |  |
|  | **OPV-naïve; 4 IPV Doses** | | | | | | |  | **OPV-naïve; 4 IPV Doses** | | | | | | |
| **Variable** | **N** | **Mean** | **Median** | **SD** | **SE** | **Min** | **Max** | **Variable** |  |  |  |  |  |  |  |
| **Overall Duration** | 8 | 8 | 6 | 7 | 2 | 3 | 24 | **Overall Duration** |  |  |  |  |  |  |  |
| **OPV-1 Duration** | 3 | 5 | 3 | 4 | 2 | 3 | 9 | **OPV-1 Duration** |  |  |  |  |  |  |  |
| **OPV-2 Duration** | 7 | 8.3 | 6 | 7 | 3 | 4 | 24 | **OPV-2 Duration** |  |  |  |  |  |  |  |
| **OPV-3 Duration** | 5 | 13 | 9 | 7 | 2 | 3 | 23 | **OPV-3 Duration** |  |  |  |  |  |  |  |
|  | **OPV-exposed; 3 IPV Doses** | | | | | | |  | **OPV-exposed; 3 IPV Doses** | | | | | | |
| **Variable** | **N** | **Mean** | **Median** | **SD** | **SE** | **Min** | **Max** | **Variable** |  |  |  |  |  |  |  |
| **Overall Duration** | 16 | 10.6 | 8 | 8 | 2 | 3 | 27 | **Overall Duration** |  |  |  |  |  |  |  |
| **OPV-1 Duration** | 10 | 7.3 | 6.5 | 4 | 1 | 3 | 14 | **OPV-1 Duration** |  |  |  |  |  |  |  |
| **OPV-2 Duration** | 11 | 7.7 | 6 | 7 | 2 | 3 | 27 | **OPV-2 Duration** |  |  |  |  |  |  |  |
| **OPV-3 Duration** | 7 | 11.3 | 9 | 9 | 4 | 3 | 27 | **OPV-3 Duration** |  |  |  |  |  |  |  |
|  | **OPV-exposed; 4 IPV Doses** | | | | | | |  | **OPV-exposed; 4 IPV Doses** | | | | | | |
| **Variable** | **N** | **Mean** | **Median** | **SD** | **SE** | **Min** | **Max** | **Variable** |  |  |  |  |  |  |  |
| **Overall Duration** | 54 | 6.7 | 6 | 5 | 1 | 2 | 24 | **Overall Duration** |  |  |  |  |  |  |  |
| **OPV-1 Duration** | 27 | 5.1 | 6 | 3 | 1 | 2 | 13 | **OPV-1 Duration** |  |  |  |  |  |  |  |
| **OPV-2 Duration** | 37 | 5.1 | 3 | 3 | 1 | 2 | 14 | **OPV-2 Duration** |  |  |  |  |  |  |  |
| **OPV-3 Duration** | 21 | 6.7 | 6 | 3 | 1 | 2 | 13 | **OPV-3 Duration** |  |  |  |  |  |  |  |
|  |  |  |  |  |  |  |  |  |  |  |  |  |  |  |  |
